# Bursoscopic Ultrasound-Guided Ossicle Resection for Osgood–Schlatter Disease

**DOI:** 10.1016/j.eats.2021.12.043

**Published:** 2022-04-22

**Authors:** Kentaro Fujita, Junsuke Nakase, Rikuto Yoshimizu, Mitsuhiro Kimura, Tomoyuki Kanayama, Hiroyuki Tsuchiya

**Affiliations:** Department of Orthopaedic Surgery, Graduate School of Medical Sciences, Kanazawa University, Japan

## Abstract

Osgood–Schlatter disease commonly affects physically active adolescents. It is a common cause of anterior knee pain and inflammation in this population. Its symptoms typically subside with conservative therapy. Surgery, including resection of mobile ossicles, is considered when the pain persists on kneeling or during sports after the skeletal maturity. In this procedure, we use a direct bursoscopic approach with ultrasound-guided ossicle resection. In comparison with the classical arthroscopic approach, the bursoscopic approach uses more distally placed portals. These reduce the risk of damage to the fat pad, meniscus, and ligament. Endoscopic surgeries, including arthroscopic and bursoscopic surgeries, use intraoperative fluoroscopy to resect ossicles because the ossicle cannot be clearly identified by endoscopic imaging alone. Fluoroscopy exposes patients and surgeons to radiation. Ultrasound-guided surgery identifies the exact positional relationship between the ossicle and grasping forceps without radiation exposure since fluoroscopy is unnecessary. Moreover, the risk of residual ossicles is reduced because tiny ossicles, which are difficult to detect under fluoroscopy, are visible on ultrasound. Ultrasound-guided ossicle resection was a viable treatment option for Osgood–Schlatter disease because it eliminated radiation exposure and reduced the risk of missed ossicles.

Osgood–Schlatter disease (OSD) is a painful epiphyseal condition of the tibial tuberosity first reported in 1903.^1^^,^^2^ It most frequently affects physically active adolescents. It is one of the most common causes of anterior knee pain in this patient population. OSD refers to the traction apophysitis of the tibial tuberosity, caused by quadriceps strain. Approximately 10% of adolescents have OSD.^3^ It is mainly treated conservatively. If symptoms persist after the skeletal maturity, surgery may be indicated. Surgical procedures include open, arthroscopic, and bursoscopic surgeries. They may involve excision of any mobile ossicles and debridement of the tibial tuberosity.^4^ Endoscopic surgeries including arthroscopic and bursoscopic surgeries use intraoperative fluoroscopy because it is difficult to identify the ossicles, which are covered by bursa in endoscopic view alone. Thus, the patient is exposed to radiation. Even in using intraoperative fluoroscopy, it is difficult to evaluate the exact position of ossicles in real time, because we must check fluoroscopic images on frontal and lateral view. However, ultrasound-guided ossicle resection eliminates radiation exposure and enables evaluation of the exact ossicles position in real time. We report a successful treatment of OSD via bursoscopic ultrasound-guided ossicle resection.

## Surgical Technique (With Video Illustration)

**Fig 1:**
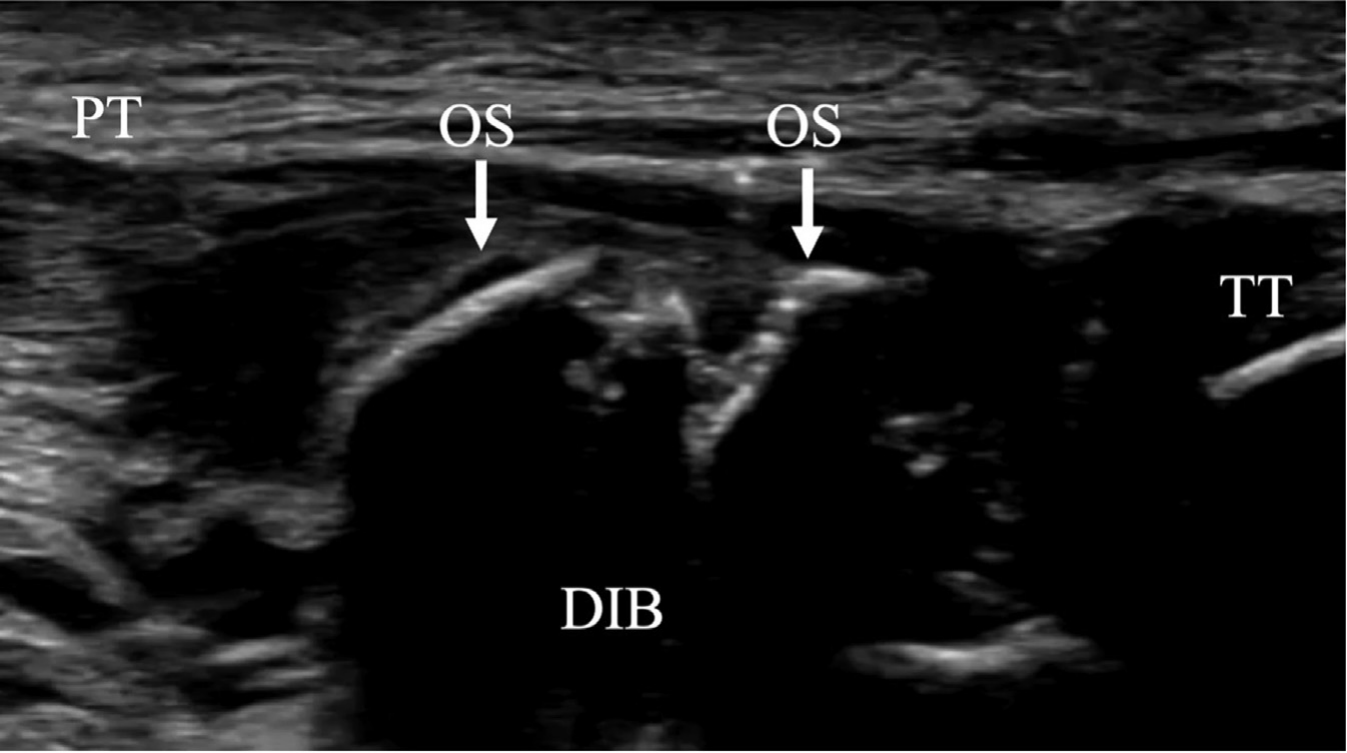
Preoperative ultrasonographic finding (long-axis image). Two ossicles are in the deep infrapatellar bursa. (DIB, deep infrapatellar bursa; OS, ossicle; PT, patellar tendon; TT, tibial tuberosity.)

**Fig 2:**
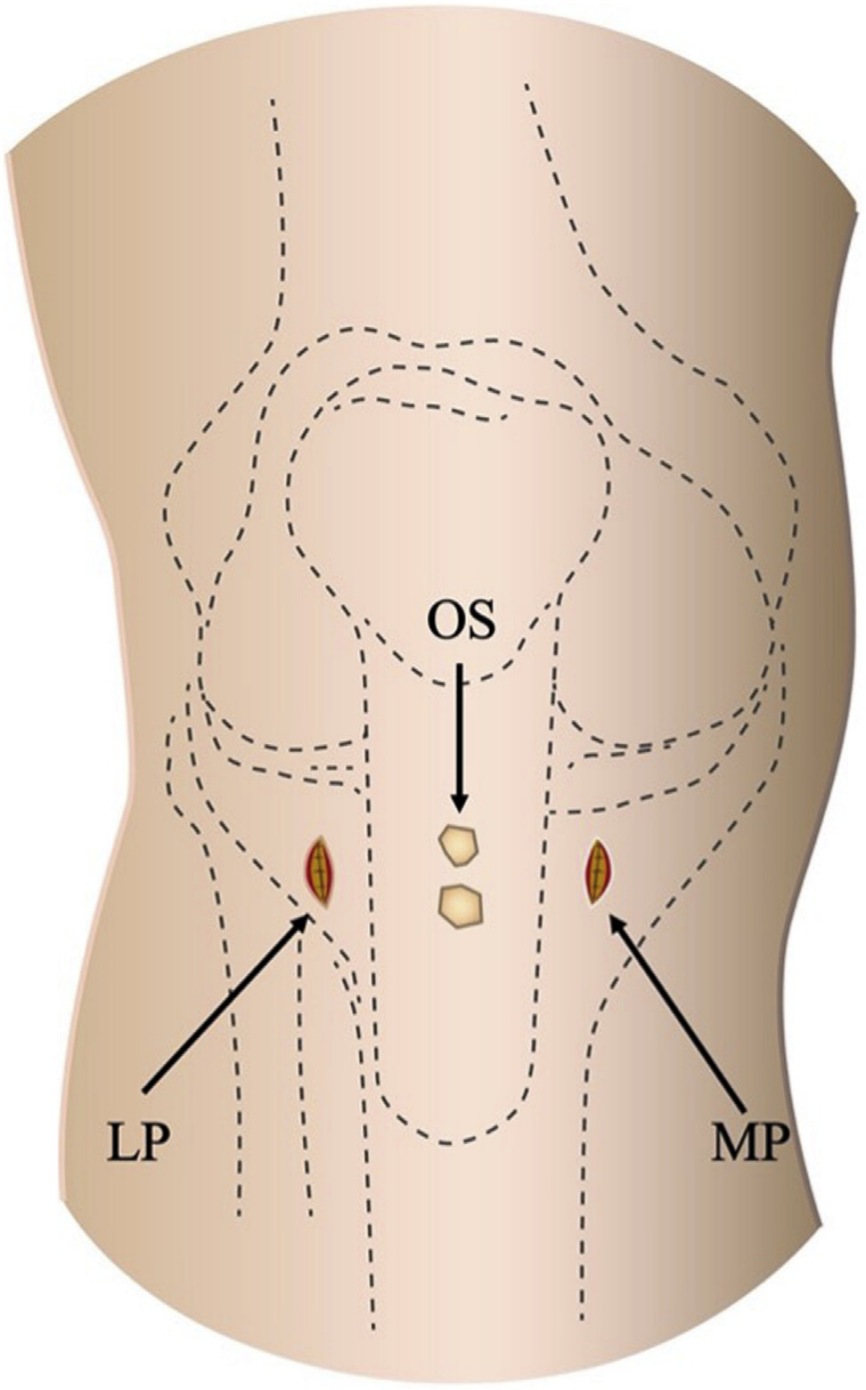
Medial and lateral portals are located at the level of the ossicles. (LP, lateral portal; MP, medial portal; OS, ossicle.)

**Fig 3:**
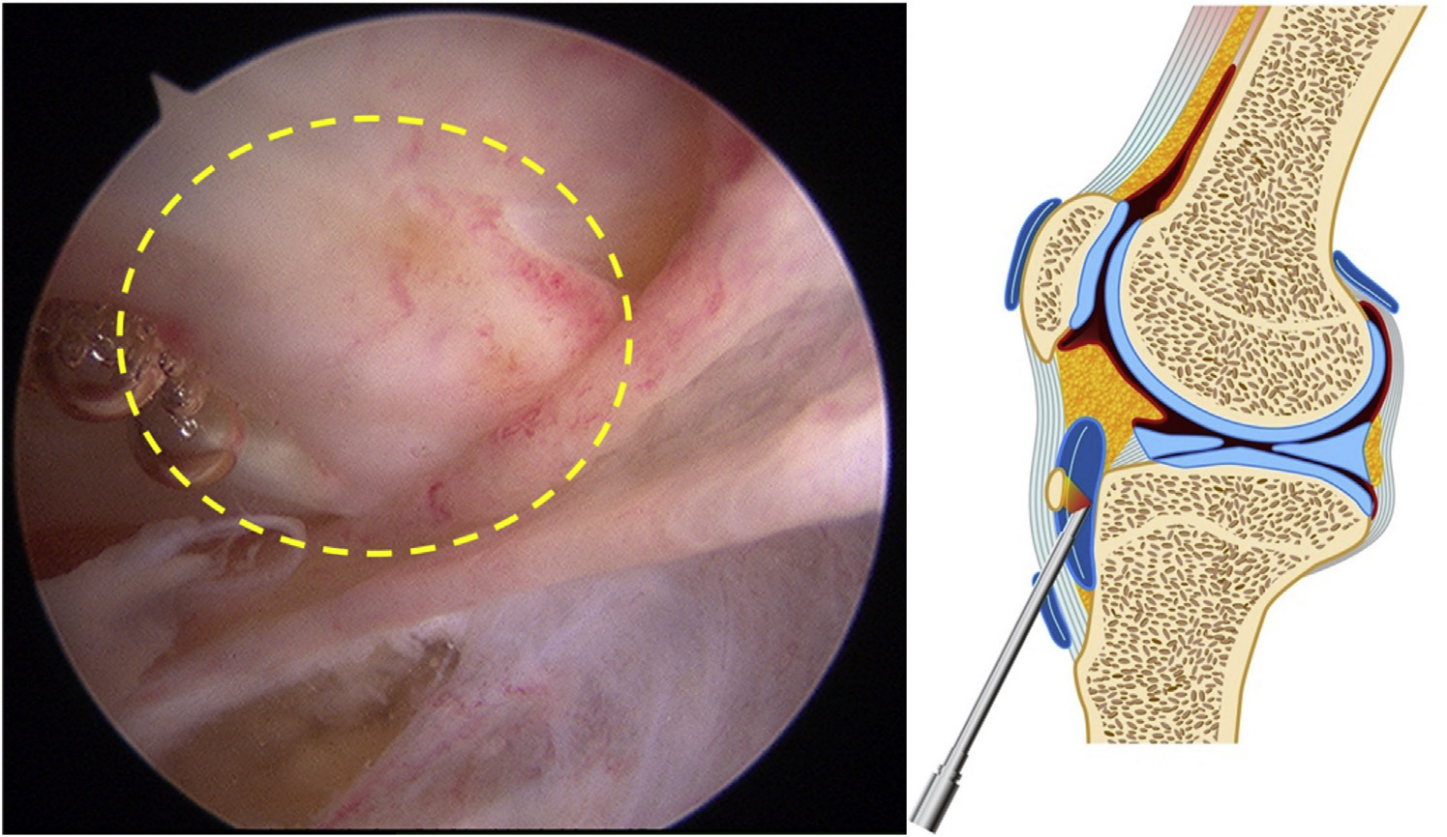
Bursoscopic view of the patient’s left knee. A 30° arthroscope is inserted into the deep infrapatellar bursa through the lateral portal with the knee extension. The area circled by the dotted line is suspected to contain an ossicle. The ossicle is difficult to appreciate via endoscopy.

**Fig 4:**
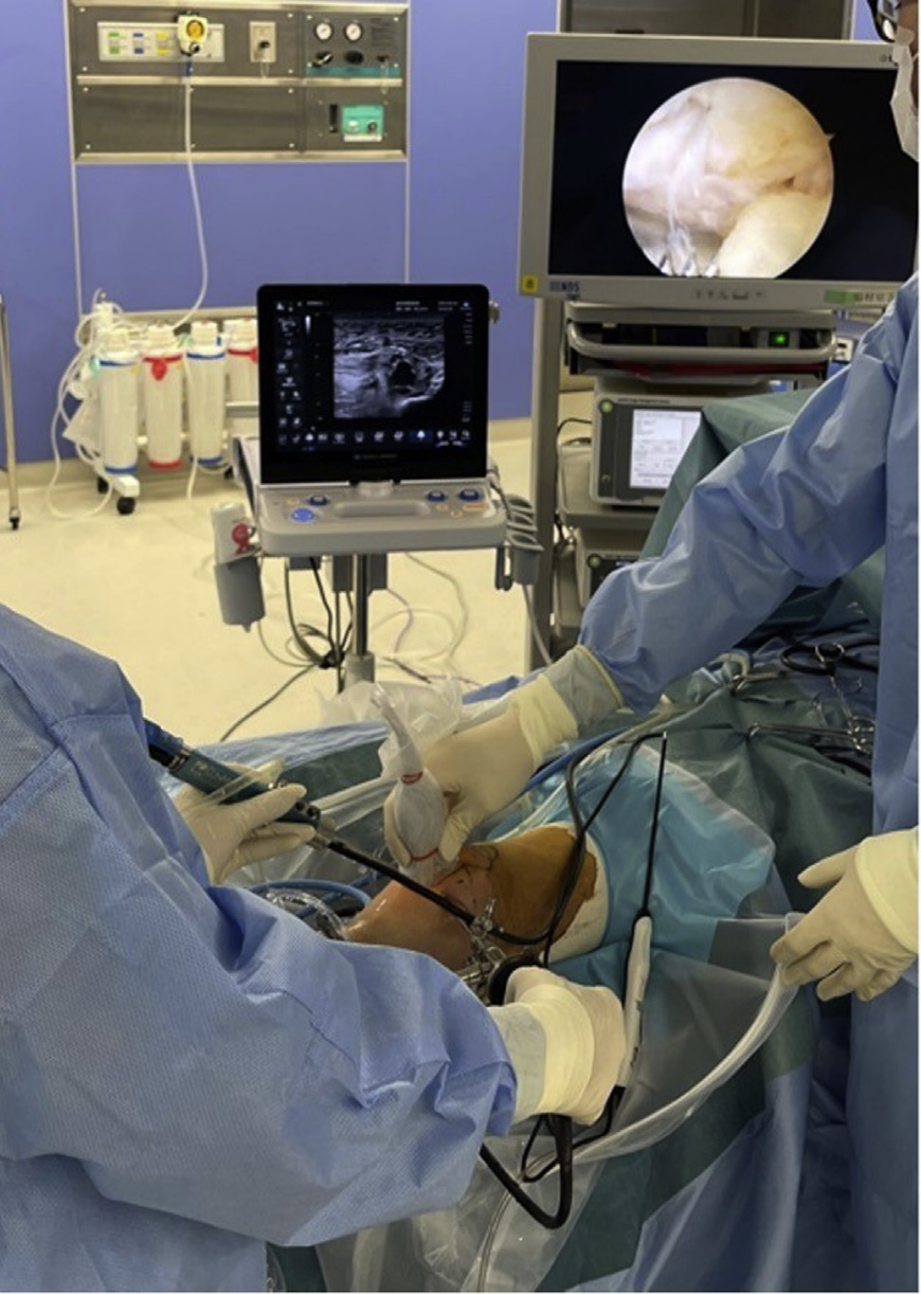
A situation in which the ossicle is being searched for using ultrasound. Intraoperative ultrasound imaging is performed by the surgical assistant. The surgeon identifies the ossicle by using an arthroscopic probe while looking at the ultrasonic monitor.

**Fig 5:**
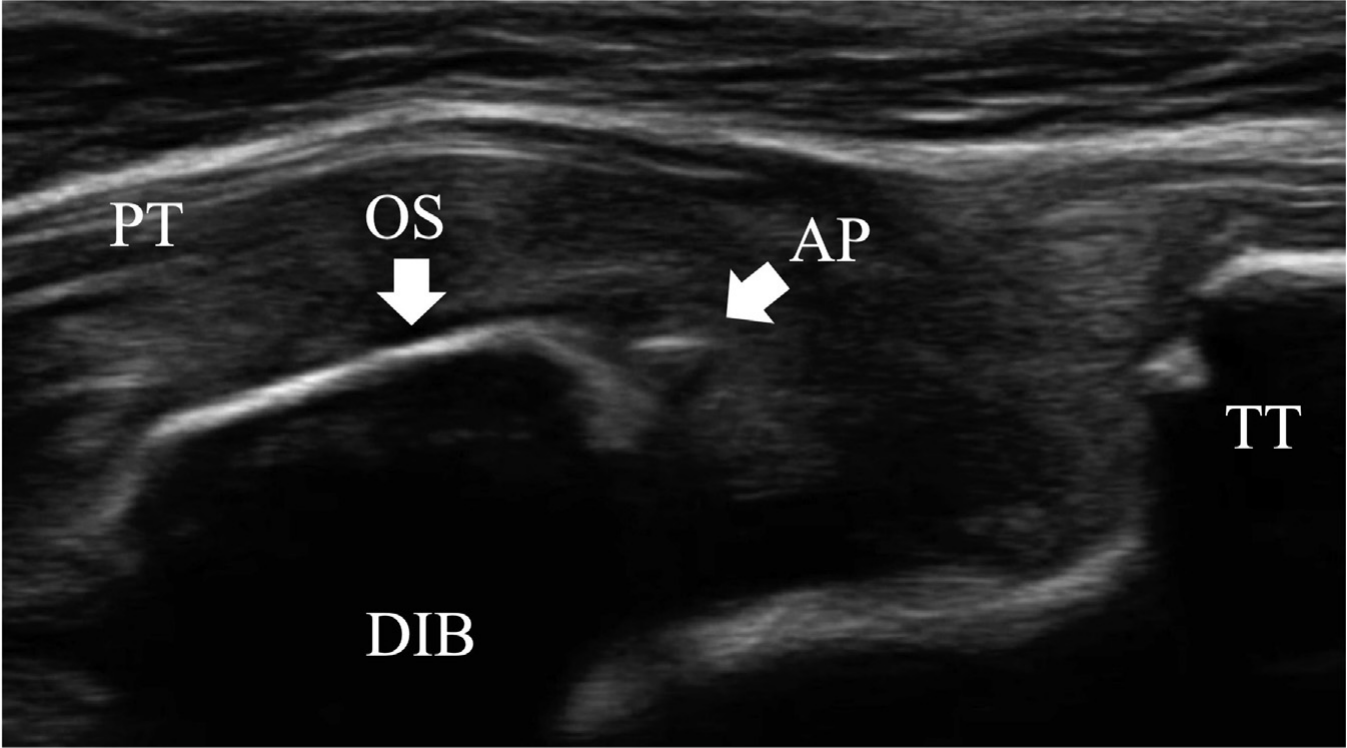
Ultrasound image while touching an ossicle with an arthroscopic probe. The ossicle is identified by touching it with an arthroscopic probe in the ultrasound image. (AP, arthroscopic device; DIB, deep infrapatellar bursa; OS, ossicle; PT, patellar tendon; TT, tibial tuberosity.)

**Fig 6:**
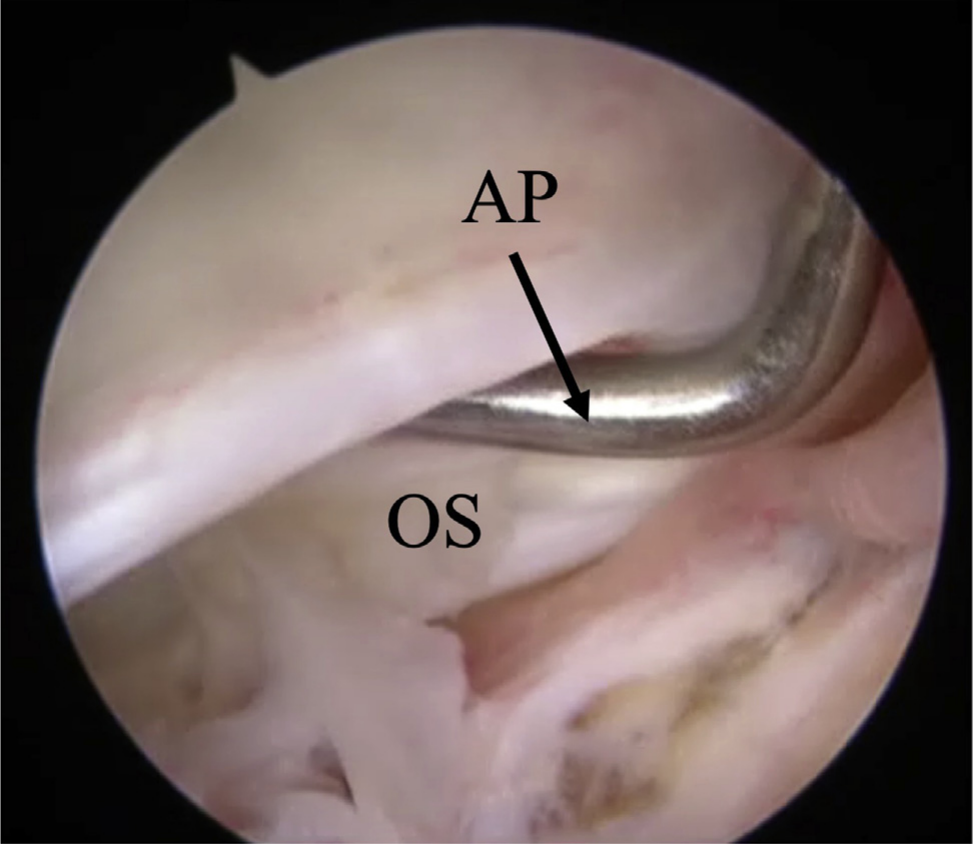
Bursoscopic view of the patient’s left knee during touching an ossicle with an arthroscopic probe. (AP, arthroscopic device; OS, ossicle.)

**Fig 7:**
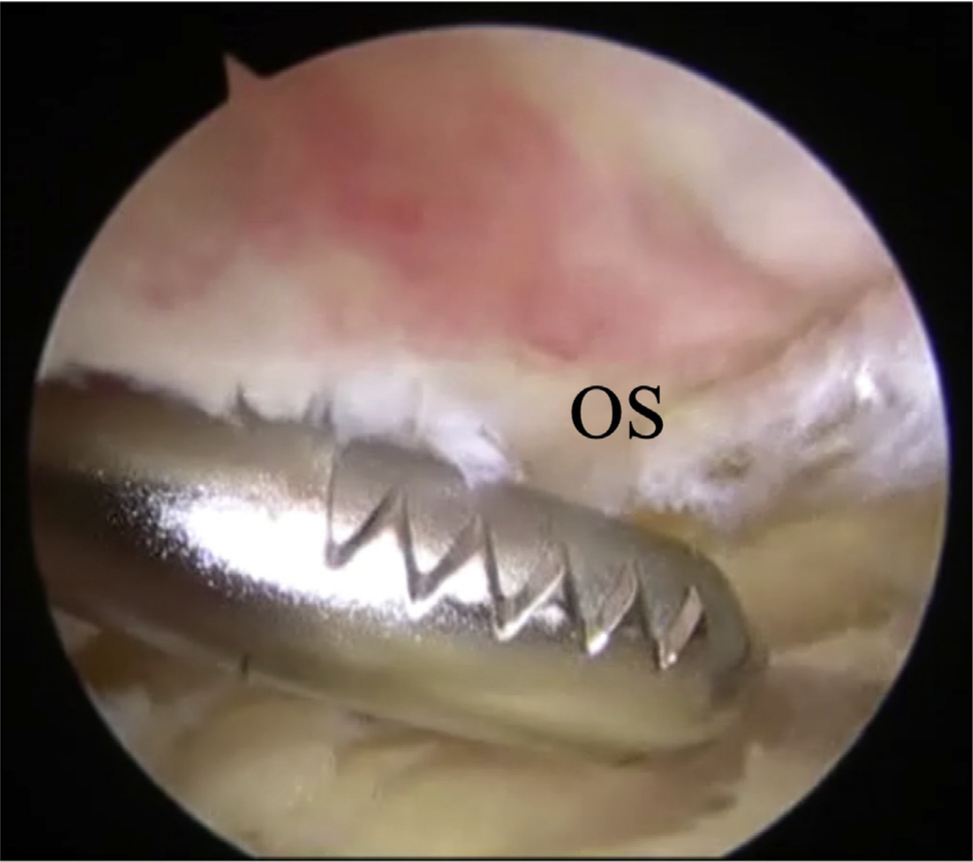
Bursoscopic view of the patient’s left knee. A 3.5-mm shaver is introduced through the medial portal to resect the bursa around the ossicle. (OS, ossicle.)

**Fig 8:**
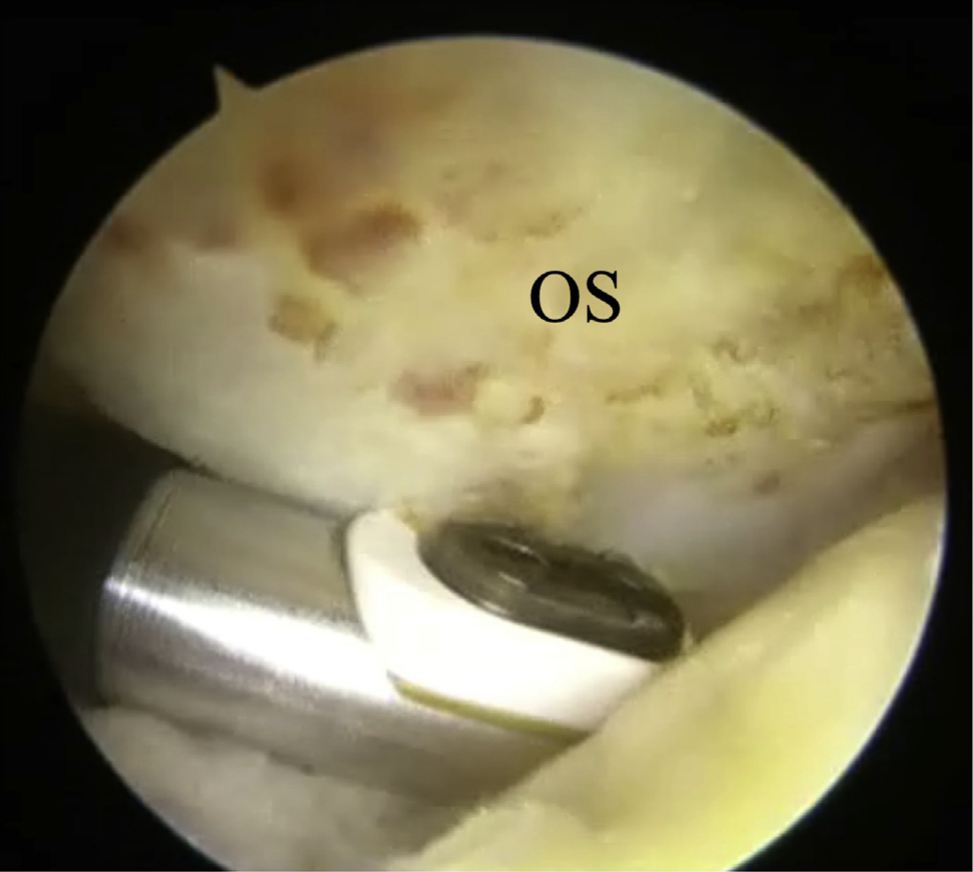
Bursoscopic view of the patient’s left knee. The ossicle is peeled out of the bursa with a radiofrequency device. (OS, ossicle.)

**Fig 9:**
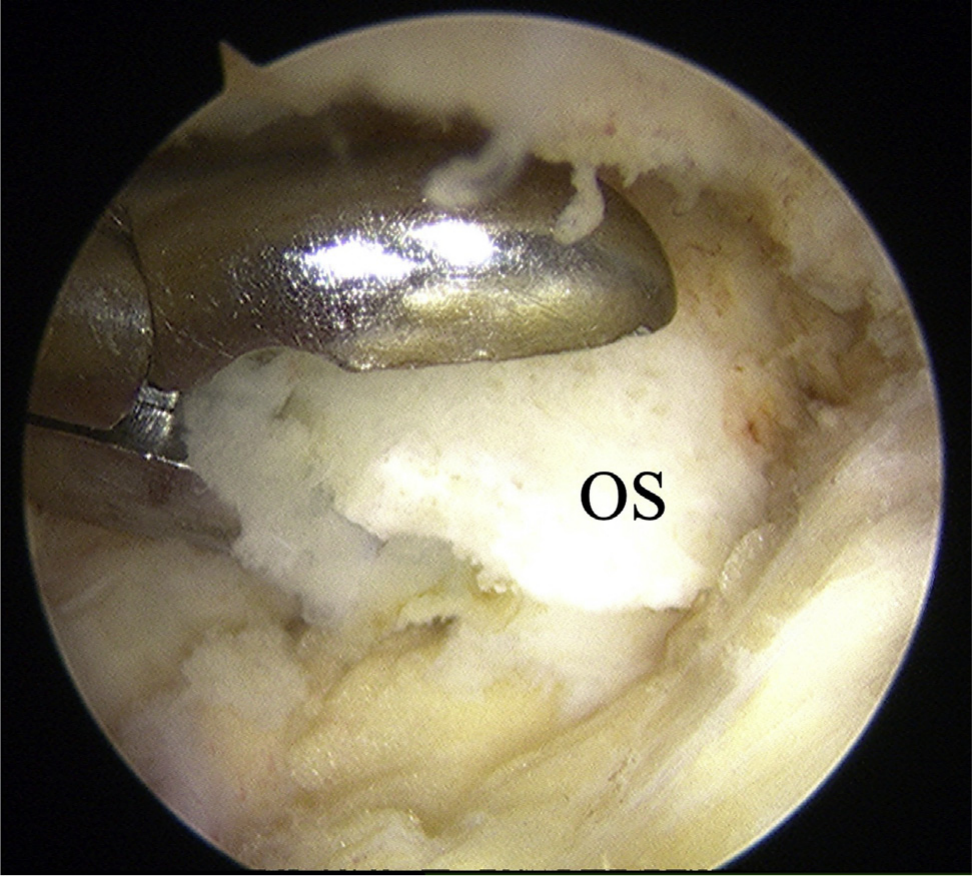
Bursoscopic view of the patient’s left knee. The ossicle is removed with forceps. (OS, ossicle.)

**Fig 10:**
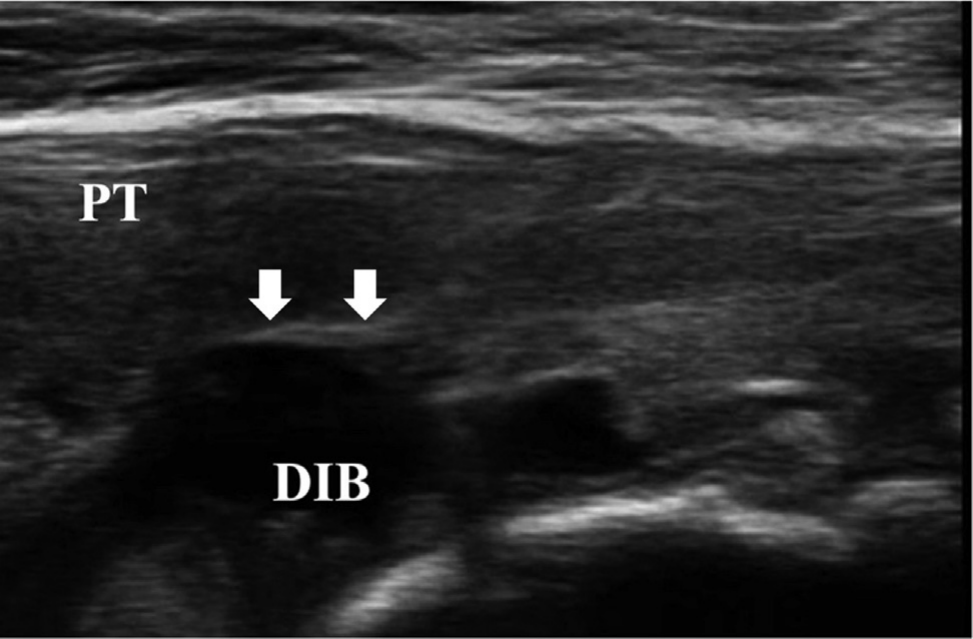
Ultrasound image of the patient’s left knee after ossicle removal. The area is inspected again by ultrasound to confirm the absence of remnant ossicles, The white arrows indicate where the ossicle is located. (DIB, deep infrapatellar bursa; PT, patellar tendon.)

The surgical technique is shown in Video 1. The patient is positioned supine on the operating table with a thigh tourniquet applied. The location of the ossicle is confirmed using ultrasound B-mode imaging (SONIMAGE HS1; KONICA MINOLTA, Tokyo Japan) (Fig 1). Portals are designated at the 1 cm away from medial and lateral edges of the patellar tendon at the level of the ossicle (Fig 2). After the operating limb is sterilized and draped, a sterile cover is placed over the ultrasonic probe, located on contralateral side of the patient. This placement allows the surgeon to see both the arthroscopic and the ultrasound monitors simultaneously. Ten milliliters of 1% lidocaine and 10 mL of saline are injected into the deep infrapatellar bursa to mitigate pain and maintain the working space under ultrasound-guided using in-plane technique. A 30° arthroscope (DYONICS; Smith & Nephew, Andover, MA) is inserted into the deep infrapatellar bursa through the lateral portal with the knee extension. The ossicles are identified via ultrasound because they are difficult to appreciate via endoscopy (Figs 3 and 4). The ossicle is identified by touching it with an arthroscopic probe in the ultrasound image (Figs 5 and 6). Following identification, a 3.5-mm shaver is introduced through the medial portal to resect the bursa around the ossicle (Fig 7). The ossicle is peeled out of the bursa with a radiofrequency device (Synergy; Arthrex; Tokyo, Japan) (Fig 8) and removed with forceps (Fig 9). After ossicle removal, the area is inspected again by ultrasound to confirm the absence of remnant ossicles (Fig 10), and the incision is closed. The patient is allowed to bear full weight with a full range of motion immediately after surgery. Jogging is permitted 2 weeks postoperatively, whereas strenuous activity is permitted 6 weeks postoperatively.

## Discussion

OSD is mainly treated conservatively. This involves rest, physical therapy, medication, proper padding of tibial tubercle.^5^ In most cases provides, conservative treatment provided symptomatic relief.^6^ In contrast, it is unresolved OSD when the fragment of secondary ossification center of the tibial tuberosity has finished ossification and the cause of pain is due to deep infrapatellar bursitis. Even in patients with unresolved OSD, ultrasound-guided injection of lidocaine and steroid into deep infrapatellar bursa improves symptoms in most patients. Particularly in highly active patients with unresolved OSD, deep infrapatellar bursitis occurs repeatedly and symptoms such as pain on kneeling may continue. In such cases, conservative therapy is ineffective and surgical procedure may be indicated.

Previous reports have classified surgical techniques as open or arthroscopic surgery.^5^ The disadvantage of open surgery is the large number of incisions. Meanwhile, arthroscopic surgery has been associated with several advantages, including small incisions, minimal wound pain and scarring, and accelerated functional recovery.^7^ However, its disadvantages include possible damage to the infrapatellar fat pad, anterior horn of the meniscus, and intermeniscal ligament.^8^ In addition to open and arthroscopic surgery, bursoscopic surgery also has become available.^4^ In comparison with classical arthroscopy, bursoscopy uses more distally placed portals. These portals reduce the risk of damage to the infrapatellar fat pad, anterior horn of the meniscus, and intermeniscal ligament.^9^ Bursoscopic surgery also facilitates the easy removal of ossicles, located distal to the deep infrapatellar bursa. One potential disadvantage of bursoscopic surgery, especially in the debridement of the tibial tuberosity,^4^ is the limited working space. However, debridement is rarely performed, and the provided space is sufficient for ossicle resection.

**Fig 11:**
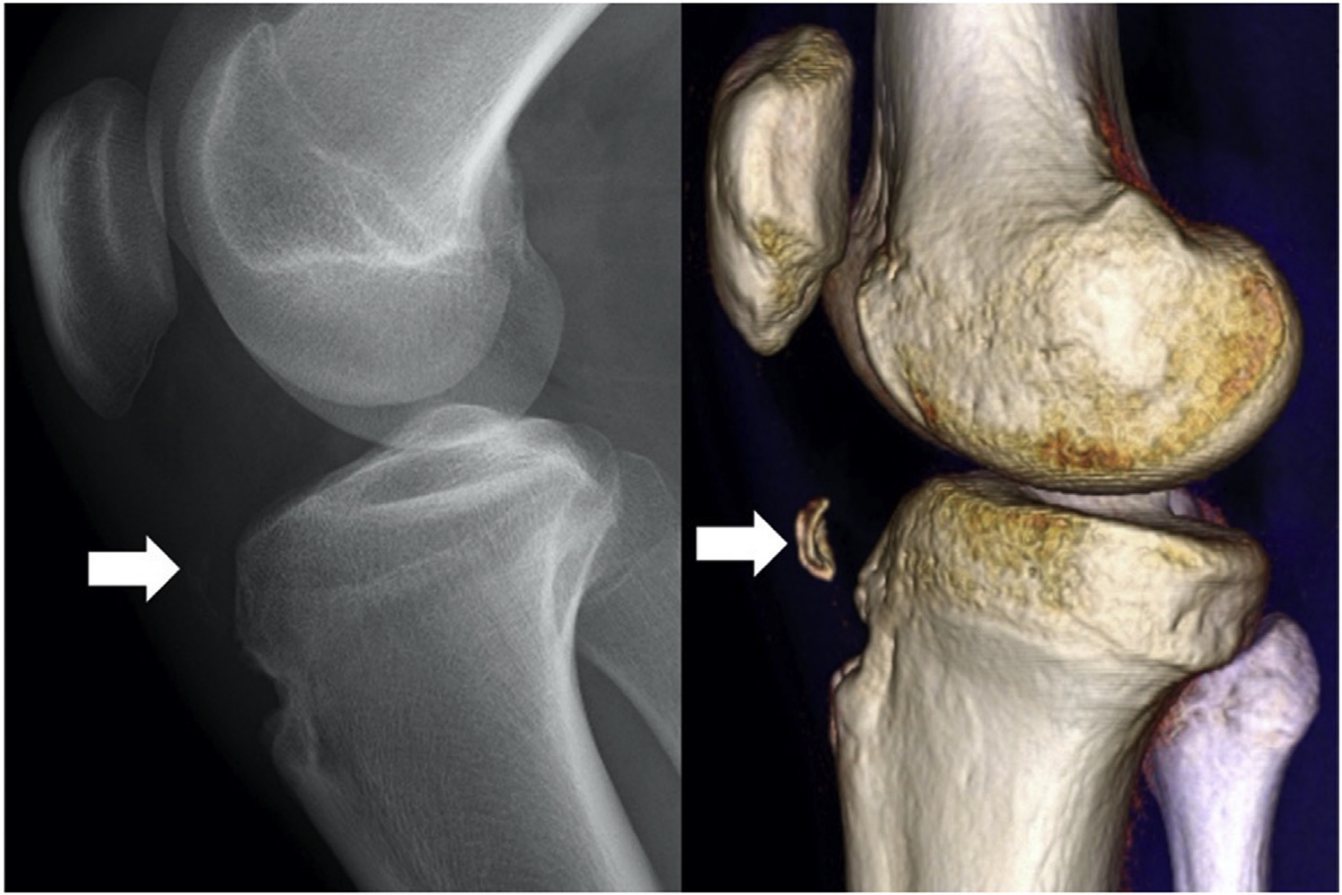
View on lateral radiograph (A) and 3-dimensional CT scan (B). The ossicle is shown (arrow). The ossicle may be difficult to identify plain radiographic images.

Ultrasound-guided ossicle resection has several advantages. First, it eliminates radiation exposure. Previous bursoscopic and arthroscopic surgeries used intraoperative fluoroscopy for ossicle resection. Thus, the patients, surgeons, anesthesiologists, and paramedical staff were exposed to radiation. Second, this procedure accurately identifies tiny ossicles, reducing the risk of remnant ossicles. Compared with the limited view of endoscopy, ultrasound imaging accurately depicts the surgical field and identifies small ossicles (Fig 11). Furthermore, ultrasound imaging enables to visualize soft tissues as well, leading to less damage to patellar tendon. All advantages and disadvantages and pearls and pitfalls are presented in Tables 1 and 2.

**Table 1:** Pearls and Pitfalls

Pearls	• The location of the ossicle is confirmed using the ultrasound before operating limb is sterilized. • The ossicles are identified via ultrasound because they are difficult to appreciate via endoscopy and fluoroscopy. • After ossicle removal, the area is inspected again by ultrasound to confirm the absence of remnant ossicles.
Pitfalls	• Intraoperative ultrasound imaging is performed by the surgical assistant. • During the ossicle resection, be careful to avoid damage to the patellar tendon.

**Table 2:** Advantages and Disadvantages

Advantages	• The risk of damage to the patellar tendon, infrapatellar fat pad, anterior horn of the meniscus, and intermeniscal ligament is reduced. • There is no radiation exposure in this surgical procedure. • The risk of presence of remnant ossicles is reduced.
Disadvantage	• Working space is limited • This surgical procedure requires a surgical assistant who is able to perform ultrasound imaging.

In conclusion, bursoscopic ultrasound-guided ossicle resection might be a viable alternative to intraoperative fluoroscopy for OSD because it eliminates radiation exposure and accurately identifies tiny ossicles.

## Supplementary Data

ICMJE author disclosure forms

Video 1 The patient is positioned supine on the operating table. We marked the ossicle location using the ultrasound B-mode. The medial and lateral portals are located at the medial and lateral edges of the patellar tendon at each marked ossicle level. A 30° arthroscope is inserted into the deep infrapatellar bursa through the lateral portal, and ultrasound is used to explore the area and identify the ossicle. The bursa near the ossicle is resected using an arthroscopic shaver through the medial portal. The ossicle is peeled off with a radiofrequency device and removed using forceps. After the ossicle excision, ultrasound exploration is repeated to confirm no remaining ossicles, and the incision is closed.
